# Proteomic analysis to define predictors of treatment response to adalimumab or methotrexate in rheumatoid arthritis patients

**DOI:** 10.1038/s41397-019-0139-4

**Published:** 2019-12-10

**Authors:** Stephanie F. Ling, Nisha Nair, Suzanne M. M. Verstappen, Anne Barton, Hans-Dieter Zucht, Petra Budde, Peter Schulz-Knappe, MATURA consortium, Darren Plant

**Affiliations:** 1 Centre for Genetics and Genomics Versus Arthritis, Centre for Musculoskeletal Research, The University of Manchester, Manchester, UK; 2 NIHR Manchester Biomedical Research Centre, Manchester University NHS Foundation Trust, Manchester Academic Health Science Centre, Manchester, UK; 3 Centre for Epidemiology Versus Arthritis, Centre for Musculoskeletal Research, The University of Manchester, Manchester, UK; 4 Formerly, Protagen AG, Dortmund, Germany; 5 Oncimmune Germany GmbH, Dortmund, Germany

**Keywords:** Predictive medicine, Predictive markers

## Abstract

Seropositivity for anti-citrullinated peptide antibodies (ACPA) in patients with rheumatoid arthritis (RA), a chronic autoimmune arthritis, is associated with worse long-term disease outcomes. ACPA is ubiquitously tested in RA patients, but other autoantibodies exist (in both citrullinated and non-citrullinated form) which may provide additional information on RA subtypes and/or treatment response. We used a multiplex bead-based assay of 376 autoantibodies to test associations between these autoantibodies and treatment response in RA patients. Clusters of patients with similar autoantibody expression were defined and cluster membership was associated with treatment response. Thirty-four autoantibodies were differentially expressed in RA patients compared with healthy controls; citrullinated vimentin was associated with treatment response. A selection of citrullinated autoantibodies was found to be associated with treatment response in a subanalysis of ACPA-negative RA patients. Finer ACPA specificities in ACPA-negative RA patients may be predictive of treatment response and could represent a rich vein of future study.

## Introduction

Rheumatoid arthritis (RA) is a chronic autoimmune arthritis that can also have multisystem involvement. RA pathogenesis involves numerous processes, including autoreactivity of T cells and autoantibody formation [1]. Testing of two of these autoantibodies, rheumatoid factor (RF) and anti-citrullinated peptide antibodies (ACPA), aid both diagnosis and prognosis and form part of both European and British guidelines for management [2, 3]. In the healthy population, ACPA was found to be positive in 0.8% of a population of 40,136 individuals in a large Dutch study [4], and RF has been estimated as positive in 5% of healthy 50-year-olds and 10–25% of healthy 70-year-olds [5]. Seropositivity for ACPA is associated with worse long-term disease outcomes in RA [6], and it is important to achieve clinical remission in these patients as quickly as possible to prevent joint damage and disability. In addition, RF and ACPA seropositivity are associated with reduced response to anti-TNF drugs (a class of biological disease-modifying anti-rheumatic drug, bDMARD) [7].

Whilst RF and ACPA are known to be prognostic indicators in RA, those patients who are seronegative for RF/ACPA are considered to be less at risk of a severe disease course. For example, in a primary care inception cohort of inflammatory arthritis patients recruited between 1990 and 1994, it was found that ACPA-positive patients had less benefit from treatment than ACPA-negative patients [8]. More recently, however, RF seronegativity was associated with nonresponse to methotrexate (a conventional synthetic DMARD, csDMARD) in a cohort of bDMARD-naïve patients [9].

Although ACPA and RF are the most investigated autoantibodies and are included in criteria for RA, other autoantibodies have been associated with RA prognosis, such as anti-carbamylated protein (anti-CarP) antibodies, which have been found to be associated with a more severe clinical course in RA patients who are seronegative for ACPA [10]. In addition, the search for predictors of prognosis and/or treatment response in RA patients remains an area of development, particularly in those who are RF/ACPA seronegative. Most previous studies have used the commercial CCP2 assay to classify ACPA positivity, but other autoantibodies, both citrullinated and non-citrullinated exist, which may provide additional information on subtypes of RA and/or treatment response.

We used a multiplex bead-based approach to analyse sera from healthy controls (HC) and RA patients to determine: (1) similarities between RA patients in autoantibody expression and whether co-expression profiles are related to treatment outcomes; (2) differentially expressed autoantibodies between RA patients and HC; and (3) whether any of the autoantibodies more frequently expressed in RA patients were associated with treatment response.

## Materials/Subjects and methods

### Study subjects

HC were chosen from specimens from blood donors of the Bavarian Red Cross, Germany [11]. RA patients were recruited from two cohorts. Patients with early RA commencing methotrexate were recruited from the Rheumatoid Arthritis Medication Study (RAMS; approving ethics committee: Central Manchester Research Ethics Committee (now NRES Committee North West – Greater Manchester Central; REC reference number: 08/H1008/25), a UK-based multi-centre prospective study [9]. Patients were aged 18 years or over with a physician diagnosis of either RA or undifferentiated polyarthritis and were commencing methotrexate for the first time, either as monotherapy or in combination with other csDMARDs or oral steroids. Patients with current or previous exposure to a bDMARD were ineligible for recruitment. Patients with established RA commencing adalimumab, an anti-TNF bDMARD, were recruited from the Biologics in RA Genetics and Genomics Study Syndicate (BRAGGSS; approving ethics committee: Central Manchester Research Ethics Committee (now NRES Committee North West – Greater Manchester Central; REC reference number: 04/Q1403/37), a large UK-based multi-centre prospective study [7]. Patients were Caucasian and aged 18 years or over, fulfilling the American College of Rheumatology 1987 revised criteria for the classification of RA [12] and with Disease Activity Score in 28 joints using C-reactive protein (DAS28-CRP) ≥ 5.1 (indicative of active disease) [13] despite treatment with two previous csDMARDs. Informed consent was obtained from all subjects.

Serum samples were collected at baseline in both RA cohorts. Serum samples from HC and RA were processed and stored by Protagen AG, Dortmund, Germany for autoantibody profiling. RA serum samples were sent to the Centre for Musculoskeletal Research, The University of Manchester, Manchester, UK for processing, storage and analysis. ACPA was measured on RA samples using a commercially available ELISA (CCP2, Axis-Shield Diagnostics Ltd, Dundee, UK).

Demographic and clinical data were obtained at baseline. For the purpose of this study, DAS28-CRP measured at 3 months in BRAGGSS patients and at 6 months in RAMS patients was used. Therapeutic response was defined as good, moderate or poor according to the European League Against Rheumatism (EULAR) response criteria [14] at these timepoints.

### Measurement of autoantibodies

Bead-based antigen arrays were used for the multiplex analysis of IgG autoantibody reactivity against 376 recombinant human protein antigens associated with autoimmune disease to detect autoantibodies. Overall, 39 of the 376 autoantigens were in citrullinated form. The full list of antigens is provided in the Supplementary Information. Detailed methodology for protein expression and multiplex autoantibody measurement has previously been described [15]. In brief, antigens were produced in *E. coli*, purified and covalently coupled to magnetic carboxylated colour-coded beads (MagPlex^TM^ microspheres, Luminex Corporation, Austin, Texas). Antigen-coupled beads were combined, incubated with probands’ sera and after washing procedures, incubated with a secondary PE-labelled anti-human IgG antibody. The beads were washed again, then analysed in a FlexMap3D instrument (Luminex Corporation). The median fluorescence intensity (MFI) values, reflecting semi-quantitative autoantibody levels, were obtained for each colour-coded antigen-coupled bead and each sample. This procedure was carried out once for each sample.

### Statistical analysis

MFI values for HC and RA were normalised and log2-transformed. The 95th percentile for each autoantibody in HC was used to determine whether an RA sample was positive/negative for that autoantibody. Proteins with <10% frequency in RA patients were subsequently excluded from analysis.

Correlation analysis of autoantibody data using the Pearson method was carried out in RA patients to define clusters of patients with similar autoantibody profiles (co-prevalence analysis) as described previously [16]. Logistic regression was used to determine seropositive autoantibodies associated with membership of each defined cluster vs. all other patients outside the cluster of interest; both seropositivity for autoantibodies and cluster membership were analysed as binary variables. The Benjamini–Hochberg adjustment was used to correct for multiple testing and autoantibodies with a significance level of *p* < 0.05 were retained. Associations between cluster membership and treatment outcomes at 3/6 months were analysed using: (i) linear regression for improvement in DAS28, with a negative value indicating an increased score i.e. worsening disease activity; (ii) logistic regression for good vs. moderate/poor and poor vs. moderate/good EULAR response. All regression analyses were adjusted for age, gender, disease duration and baseline DAS28.

Further analysis was carried out in a subset of patients with available ACPA data according to CCP2 assays. Linear regression was used to determine autoantibodies differing in MFI between RA and HC; *p*-values were adjusted using the Benjamini–Hochberg correction. Significant autoantibodies (adjusted *p* < 0.05) were tested for associations with treatment outcomes in RA patients only using: (i) linear regression for improvement in DAS28; (ii) logistic regression for good-/poor-vs-all EULAR response. Multivariate models consisting of measures of treatment response as the dependent variable and various autoantibody expression profiles of interest as independent variables were defined, and the Akaike information criterion (AIC) was used to compare goodness-of-fit between models. Again, all regression was adjusted for age, gender, disease duration and baseline DAS28.

All statistical analysis was carried out in R v3.4.2 (https://www.r-project.org/).

## Results

### Patient characteristics

A total of 52 HC, 150 BRAGGSS patients and 136 RAMS patients (286 RA patients in total) were included in the initial analysis; the characteristics of study participants are summarised in Table 1. ACPA status, as measured using the commercial CCP2 assay previously described, was available in 168 RA patients (37 BRAGGSS, 131 RAMS), and 90 of these 168 patients were ACPA positive (53.6%). Controls were similar to cases in age and gender, with no significant differences between the groups. Patients in the BRAGGSS cohort had longer disease duration (median 7.6 years [IQR 2.6, 7.2], compared with median 0.8 years [IQR 0.4, 1.4] in RAMS) and higher DAS28 score at baseline (mean 5.18 (SD 0.89), compared with mean 4.20 (SD1.16) in RAMS), as expected in patients commencing a bDMARD in the UK.

**Table 1:** Demographic and clinical characteristics of patients at baseline and EULAR response after 3/6 months of treatment.

*Characteristic*	HC (*n* = 52)	All RA (*n* = 286)	*p*-value (HC vs RA)	BRAGGSS (*n* = 150)	RAMS (*n* = 136)
Age (years), mean (SD)	51.0 (9.2)	59.0 (12.8)	0.12	58.0 (11.7)	60.0 (13.8)
Female sex, *n* (%)	39 (75.0%)	212 (74.1%)	1.00	111 (74.0%)	101 (74.3)
Disease duration (years), median [IQR]	–	2.1 [0.8, 9.0]	–	7.6 [2.6, 17.2]	0.8 [0.4, 1.4]
ACPA positive, *n* (%)	–	90 (53.6) [118 missing]	–	24 (64.9) [113 missing]	66 (50.4) [5 missing]
Baseline DAS28, mean (SD)	–	4.71 (1.14)	–	5.18 (0.89)	4.20 (1.16)
**EULAR response at 3 months**	–		–		
Good, *n* (%)		117 (40.9)		58 (38.7)	59 (43.4)
Moderate, *n* (%)		60 (21.0)		58 (38.7)	2 (1.5)
Poor, *n* (%)		109 (38.1)		34 (22.7)	75 (55.2)

BRAGGSS patients were treated with adalimumab for 3 months; RAMS patients were treated with methotrexate for 6 months and were bDMARD-naïve

### Co-expression analysis

**Fig. 1:**
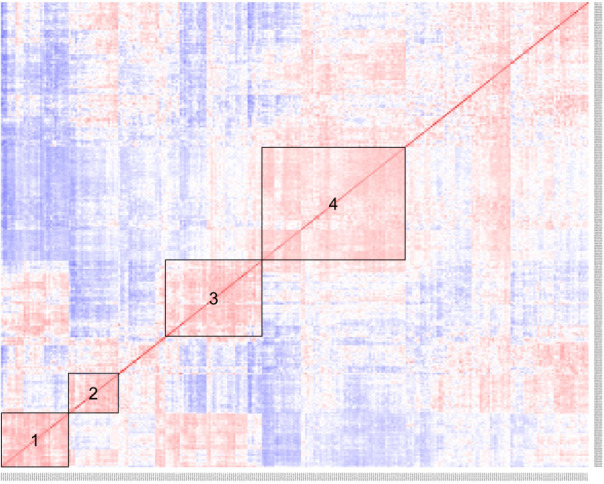
Co-prevalence heatmap displaying four distinct clusters of RA patients with similar expression of autoantibodies. Red signatures demonstrate similar seropositivity of autoantibodies, whereas blue signatures represent patients with dissimilar seropositivity of autoantibodies.

Following the exclusion of autoantibodies with <10% seropositivity in RA patients, 181 autoantibodies were retained for analysis. Four clear clusters of patients were identified from co-prevalence analysis (Fig. 1). Autoantibodies associated with membership of various clusters are detailed in Table 2. In Cluster 1 (no ACPA reactivity), 10 autoantibodies were significantly associated with cluster membership; three of these were unique to Cluster 1: cathepsin L1 (CTSL), Toll-like receptor 2 (TLR2) and interleukin (IL)–15. Cluster 2 membership was only associated with one autoantibody, complement C4-B (C4B). None of the autoantibodies associated with either Cluster 1 or Cluster 2 were in citrullinated form. Cluster 3 (moderate ACPA reactivity, cross-reactivity with Clusters 1 and 4) was associated with 11 autoantibodies, but these overlapped with those associated with either Cluster 1 (all non-citrullinated) or Cluster 4 (all citrullinated). Cluster 4 patients (high ACPA reactivity) were associated with 20 autoantibodies, 16 of which were unique to Cluster 4 (see Table 2) and all significantly associated autoantibodies were in citrullinated form. All citrullinated antibodies associated with Clusters 3 and 4 were differentially expressed in RA patients compared with controls, apart from citrullinated protein disulphide-isomerase A6 (PDIA6) in Cluster 4.

**Table 2:** Seropositive autoantibodies associated with patient membership in each cluster.

Autoantibody	OR_adj_ (95% CI)	*p*-value	Adj *p*-value
*Cluster 1 (n* *=* *33) – no ACPA reactivity*			
IL6R	36.33 (13.13–100.55)	4.59E–12	<1E–06
IL17RA	23.39 (9.53–57.39)	5.80E–12	<1E–06
CD86	19.21 (8.13–45.37)	1.58E–11	<1E–06
CSF2RA	20.08 (8.38–48.10)	1.71E–11	<1E–06
CD80	18.66 (7.87–44.21)	2.95E–11	<1E–06
CCL2	17.74 (7.52–41.86)	5.10E–11	<1E–06
TNFSF13	16.49 (7.04–38.59)	1.06E–10	<1E–06
CTSL^a^	3.44 (1.58–7.50)	1.90E–03	0.023
TLR2^a^	3.56 (1.51–8.38)	3.72E–03	0.037
IL15^a^	3.18 (1.43–7.06)	4.44E–03	0.040
*Cluster 2 (n* *=* *24) – C4B reactivity*			
C4B^a^	6.11 (2.35–15.92)	2.08E–04	0.019
*Cluster 3 (n* *=* *47) – moderate ACPA reactivity, cross-reactivity with Clusters 1 and 4*			
CSF2RA	9.50 (4.44–20.34)	6.66E–09	1E–06
CD80	9.27 (4.34–19.79)	8.86E–09	1E–06
IL17RA	8.76 (4.17–18.41)	1.03E–08	1E–06
CCL2	8.96 (4.20–19.11)	1.41E–08	1E–06
TNFSF13	9.00 (4.14–19.56)	2.86E–08	1E–06
CD86	8.60 (4.00–18.50)	3.65E–08	1E–06
IL6R	6.99 (3.43–14.25)	8.87E–0	2E–06
VIM_c	3.78 (1.89–7.59)	1.78E–04	0.004
SPP1_c	3.23 (1.64–6.36)	6.91E–04	0.014
EIF4H_c	3.25 (1.55–6.82)	1.82E–03	0.033
CLU_c	2.78 (1.45–5.36)	2.20E–03	0.036
*Cluster 4 (n* *=* *70) – high ACPA reactivity*			
SPP1_c	20.48 (9.30–45.10)	6.37E–14	<1E–06
RBMS1_c^a^	13.92 (6.97–27.80)	8.89E–14	<1E–06
VIM_c	30.17 (12.25–74.27)	1.25E–13	<1E–06
DNAJB1_c^a^	12.83 (6.47–25.42)	2.60E–13	<1E–06
HNRNPA1_c^a^	10.54 (5.55–20.02)	6.37E–13	<1E–06
FN1_c^a^	10.23 (5.43–19.30)	6.58E–13	<1E–06
TRA2B_c^a^	10.43 (5.36–20.29)	5.04E–12	<1E–06
CLU_c	9.29 (4.85–17.78)	1.69E–11	<1E–06
NONO_c^a^	17.17 (7.28–40.57)	8.55E–11	<1E–06
SFPQ_c^a^	6.92 (3.76–12.75)	5.35E–10	<1E–06
CPSF6_c^a^	6.92 (3.76–12.75)	1.74E–09	<1E–06
TNC_c^a^	5.99 (3.23–11.11)	1.36E–08	<1E–06
AEBP1_c^a^	5.12 (2.76–9.52)	2.36E–07	3E–06
EIF4H_c	130.97 (17.44–983.47)	2.15E–06	2.8E–05
ASMTL_c^a^	7.45 (10.59–581.40)	1.96E–05	2.37E–04
PDIA6_c^a^	4.74 (2.29–9.80)	2.79E–05	3.16E–04
FGB_c^a^	3.49 (1.92–6.34)	4.34E–05	4.62E–04
DDX5_c^a^	3.79 (1.97–7.26)	6.14E–05	6.17E–04
ACTB_c^a^	3.33 (1.63–6.82)	9.86E–04	0.001
SRSF7_c^a^	2.94 (1.52–5.70)	1.35E–03	0.012

^a^Unique to cluster, "_c" suffix denotes autoantibody in citrullinated form.

Results of analysis of cluster membership with change in DAS28 at 3/6 months are detailed in Table 3. Clusters 1 and 2 showed a non-significant trend towards increased DAS28 at 3/6 months (i.e. worsening disease activity). Clusters 3 and 4 showed a non-significant trend towards DAS28 improvement at 3/6 months. Due to proximity on the original co-expression heatmap, Clusters 1 and 2 were combined, as were Clusters 3 and 4. Clusters 1/2 still showed a non-significant trend towards increased DAS28, but Clusters 3/4 demonstrated a significant association with improved DAS28 at 3/6 months (coefficient 0.38, 95% confidence intervals (CI) 0.08–0.69, adjusted *R*^2^ 0.3047, *p* = 0.013). Cluster membership was not associated with good or poor EULAR response, either separately or when Clusters 1/2 and 3/4 were combined (Supplementary Table 1).

**Table 3:** Associations between cluster membership and DAS28 improvement at 3/6 months, adjusted for age, gender, disease duration and baseline DAS28.

Cluster	Coefficient (95% confidence intervals)	Adj *R*^2^	*p*-value
1	−0.36 (−0.83–0.11)	0.2949	0.133
2	−0.22 (−0.77–0.33)	0.2908	0.429
3	0.27 (−0.14–0.68)	0.2934	0.200
4	0.31 (−0.04–0.66)	0.2969	0.081
Cluster 1/2	−0.34 (−0.71–0.04)	0.2969	0.080
Cluster 3/4	0.38 (0.08–0.69)	0.3047	0.013

### Expression of autoantibodies in patients with available ACPA status

When compared with HC, 34 autoantibodies were differentially expressed in RA patients, only five of which were in a non-citrullinated form (Table 4). All autoantibodies were increased in RA, apart from one, tumour necrosis factor ligand superfamily member 13 (TNFSF13), which had reduced expression in RA patients when compared with HC.

**Table 4:** Differentially expressed autoantibodies between RA patients and healthy controls, adjusted for age and gender.

*Protein*	*Coefficient*	*95% confidence interval*	*p-value*	*Adjusted p-value (Benjamini–Hochberg correction)*
ASMTL_c	3.41	2.50–4.33	4.66E–12	<1E–07
EIF4H_c	2.72	1.94–3.51	9.09E–11	<1E–07
SPP1_c	2.05	1.43–2.67	5.53E–10	<1E–07
NONO_c	2.11	1.45–2.78	2.43E–09	<1E–07
CLU_c	1.87	1.25–2.49	1.42E–08	1E–06
VIM_c	2.52	1.67–3.37	2.46E–08	1E–06
FN1_c	1.71	1.11–2.31	6.93E–08	2E–06
CPSF6_c	2.17	1.41–2.93	7.55E–08	2E–06
TRA2B_c	1.39	0.86–1.93	7.54E–07	1.50E–05
RBMS1_c	1.61	0.99–2.24	9.58E–07	1.70E–05
ACTB_c	1.01	0.61–1.41	1.93E–06	3.20E–05
HNRNPA1_c	1.45	0.85–2.06	4.42E–06	6.70E–05
DNAJB1_c	1.38	0.76–2.01	2.24E–05	3.12E–04
TNC_c	1.18	0.62–1.74	4.92E–05	6.37E–04
FGB_c	0.92	0.45–1.39	1.64E–04	1.98E–03
SFPQ_c	1.12	0.52–1.72	3.03E–04	3.43E–03
SRSF7_c	0.77	0.35–1.19	3.57E–04	3.68E–03
TUBB_c	0.72	0.33–1.12	3.66E–04	3.68E–03
PADI4_c	0.69	0.31–1.06	4.50E–04	4.28E–03
AEBP1_c	0.91	0.36–1.46	1.39E–03	1.26E–02
DDX5_c	0.81	0.31–1.31	1.66E–03	1.43E–02
TNFSF13	−1.17	−1.94–(−0.39)	2.62E–03	2.98E–02
APOE_c	0.86	0.27–1.44	4.31E–03	3.26E–02
RBM39_c	0.61	0.20–1.02	4.32E–03	3.26E–02
IL1B	0.53	0.17–0.89	4.53E–03	3.27E–02
TUBB	0.56	0.17–0.94	4.69E–03	3.27E–02
FGA_c	0.53	0.16–0.91	5.73E–03	3.84E–02
IGF1_c	0.60	0.18–1.03	6.11E–03	3.91E–02
FEN1_c	0.66	0.19–1.12	6.40E–03	3.91E–02
ENO1_c	0.87	0.25–1.48	6.48E–03	3.91E–02
MMP2	0.48	0.13–0.82	7.05E–03	4.12E–02
OBSL1	0.49	0.13–0.84	7.73E–03	4.37E–02
HIST1H4A_c	0.48	0.13–0.83	8.32E–03	4.56E–02
PTBP1_c	0.67	0.17–1.17	9.07E–03	4.83E–02

All 34 autoantibodies were included in multivariate regression models to determine any associations with treatment outcomes at 3/6 months. Citrullinated heterogeneous nuclear ribonucleoprotein A1 (HNRNPA1) was significantly associated with DAS28 improvement (coefficient 0.69, 95% CI 0.05–1.34, *p* = 0.037, see Supplementary Table 2). No other autoantibodies were associated with DAS28 improvement. ACPA alone (as assessed by positivity on a commercial CCP2 assay) was the best predictor of DAS28 improvement in a model adjusted for age, gender, disease duration and baseline DAS28 (coefficient 0.50, 95% CI 0.11–0.91, *p* = 0.014; AIC 572.94 vs multivariate autoantibody model AIC 599.54).

**Fig. 2:**
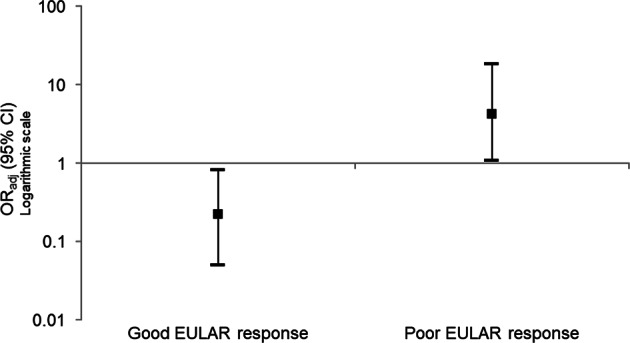
Citrullinated vimentin is associated with good and poor EULAR response at 3/6 months in RA patients with available ACPA status.

In multivariate models of all 34 autoantibodies and good and poor EULAR response (see Supplementary Tables 3 and 4, respectively), citrullinated vimentin was significantly associated with a poor EULAR response at 3/6 months, and also with reduced odds of achieving good EULAR response (OR_adj_ 4.19, 95% CI 1.07–18.32, *p* = 0.046 and OR_adj_ 0.22, 95% CI 0.05–0.81, *p* = 0.030, respectively) – see Fig. 2. A model using ACPA as measured using the CCP2 assay as the independent variable (without the 34 differentially expressed autoantibodies) was significantly associated with good EULAR response (OR_adj_ 2.40, 95% CI 1.24–4.77, *p* = 0.010), with improved model fit compared with the multivariate autoantibody model (AIC 224.07, vs AIC 267.63 in multivariate autoantibody model). ACPA was also significantly associated with reduced odds of poor EULAR response (OR_adj_ 0.40, 95% CI 0.19–0.79, *p* = 0.010), again with improved model fit (AIC 208.04 vs AIC 249.27 in multivariate autoantibody model).

### Subanalysis in ACPA-negative RA patients

**Fig. 3:**
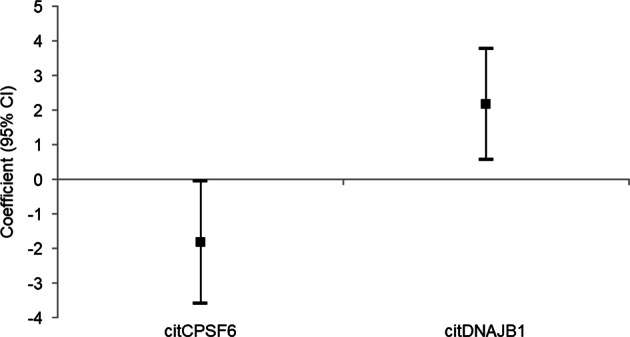
Citrullinated CPSF6 is associated with worse DAS28 at 3/6 months and citrullinated DNAJB1 is associated with improved DAS28 at 3/6 months in subanalysis of 78 ACPA-negative RA patients.

Subanalysis was carried out in the 78 ACPA-negative RA patients. Citrullinated cleavage and polyadenylation specificity factor subunit 6 (CPSF6) was significantly associated with worse DAS28 at 3/6 months (coefficient −1.83, 95% CI (−3.60)–(−0.06), *p* = 0.049; Fig. 3). In addition, citrullinated DnaJ homologue subfamily B member 1 (DNAJB1) was significantly associated with DAS28 improvement at 3/6 months (coefficient 2.17, 95% CI 0.56–3.78, *p* = 0.012). None of the 34 differentially expressed autoantibodies between RA and HC were associated with EULAR response, neither good nor poor.

## Discussion

This study used multivariate analytical techniques to interrogate a high-dimensional proteomic dataset linked to detailed clinical characteristics of RA patients. We identified distinct clusters of RA patients according to autoantibody profiles: these clusters do not appear to relate strongly to treatment response, but further exploration will be required to determine whether they are associated with other outcome measures that have not been collected in the current cohorts. We also found that testing individual autoantibodies adds nothing over the known correlation of a positive test using a commercial CCP2 assay in ACPA-positive RA patients. However, in ACPA-negative patients, a trend was observed for the association between treatment response and seropositivity of citrullinated CPSF6 and citrullinated DNAJB1; this requires replication.

In the co-expression analysis, patients who were seronegative for citrullinated forms of autoantibodies were more likely to have a worse DAS28 after 3/6 months of treatment. Conversely, patients with seropositivity for citrullinated forms of autoantibodies were more likely to have an improved DAS28. Citrullination is the result of a post-translational modification involving the conversion of the amino acid arginine into the amino acid citrulline. The citrullinated forms of autoantibodies recognised in this study are likely to represent finer specificities of ACPA beyond conventional commercially available assays, such as the CCP2 ELISA. We found that patients seropositive for finer ACPA specificities were more likely to demonstrate treatment response with either methotrexate or adalimumab. This could be because patients were treated more aggressively due to known ACPA seropositivity e.g. by more rapid escalation of methotrexate dosage or more timely escalation to bDMARD therapy. However, this has not been objectively confirmed as yet.

A total of 34 autoantibodies were found to be differentially expressed in RA patients vs. HC, and the majority of these (29/34) were in citrullinated form, which is unsurprising given the susceptibility of citrullinated proteins to an autoimmune response. Whilst two autoantibodies (citrullinated HNRNPA1 and citrullinated vimentin) were associated with DAS28 improvement after 3/6 months of treatment, seropositivity for ACPA using the commercially available CCP2 assay remained the best predictor of treatment response, so the utility of these novel biomarkers is yet to be demonstrated over established practice.

Interestingly, a proportion of ACPA-negative patients were seropositive for citrullinated autoantibodies, and this may be an important area of future development in defining predictors of treatment response in RA. The presence of autoantibodies to citrullinated CPSF6 in ACPA-negative RA patients was associated with worsening DAS28 at 3/6 months. CPSF6 is a component of the cleavage factor IM (CFIm) complex that is involved in the maturation of pre-mRNA into functional mRNA [17]. Autoantibodies to citrullinated DNAJB1 were associated with improved DAS28 at 3/6 months. DNAJB1 interacts with heat shock protein (HSP)70 and it is involved in the heat shock response [18]. Whilst these citrullinated antibodies are associated with treatment response in this cohort of RA patients, we cannot say for certain whether this relates to disease severity or is drug-specific.

Strengths of this study include recruitment of HC, meaning that analysis could be focused on autoantibodies differentially expressed by RA patients, enhancing interpretation. Furthermore, the RA patients recruited were from a well-phenotyped cohort with detailed clinical information. The RA patients were followed up over time and, therefore, longitudinal clinical information (i.e. response to medication over time) was included in analysis. However, results from the two cohorts, BRAGGSS and RAMS, were not presented separately due to sample size limitations.

In conclusion, finer ACPA specificities in ACPA-negative RA patients may be predictive of treatment response and could represent a rich vein of future study.

## Supplementary information

Appendix and supplementary data
