# Supplementary material for: Proteomic analysis to define predictors of treatment response to adalimumab or methotrexate in rheumatoid arthritis patients
Source: Pharmacogenomics J. 2019 Dec 10;20(3):516–23. doi: 10.1038/s41397-019-0139-4 (PMC7253356; doi:10.1038/s41397-019-0139-4)
Supplement: Supplementary file 1 — Appendix and supplementary data [file 41397_2019_139_MOESM1_ESM.docx]

**APPENDIX: MATURA co-investigators**

Prof Constantino Pitzalis (Queen Mary University of London)

Prof Peter Taylor (University of Oxford)

Prof Ernest Choy (Cardiff University)

Prof Iain McInnes (University of Glasgow)

Dr Mike Barnes (Queen Mary University of London)

Prof John Isaacs (Newcastle University)

Prof Christopher Buckley (University of Birmingham)

Prof Michael Ehrenstein (University College London)

Prof Peter Sasieni (Queen Mary University of London)

Prof Anne Barton (Univeristy of Manchester)

Prof Ann Morgan (University of Leeds)

Prof Gerry Wilson (University College Dublin)

Prof Paul McKeigue (University of Edinburgh)

Prof Heather Cordell (Newcastle University)

Prof Jenny Barrett (University of Leeds)

Prof Andrew Cope (Kings College London)

Prof Adam Young (University of Hertfordshire)

Prof Karim Raza (University of Birmingham)

Prof Katherine Payne (University of Manchester)

Prof Jane Worthington (University of Manchester)

Prof Deborah Symmons (University of Manchester)

Prof Kimme Hyrich (University of Manchester)

Prof Ian Bruce (University of Manchester)

Martin Hodge (Pfizer)

Anthony Rowe (Janssen)

Jeffrey Siegel (Roche/Genentech)

Michelle Mao (BGI)

Richard Watts (Qiagen)

Carolyn Cuff (Abbvie)

David Close (MedImmune)

Philippe Coterell (Avacta)

Felix Agakov (Pharmatics)

**SUPPLEMENTARY INFORMATION**

*Full list of autoantigens included on array (“_c” denotes autoantigen in citrullinated form)*

APOE, RBMS1, BGN, FGA, CXCL5, HNRNPA1, VIM, AEBP1, TRA2B, HIST1H2BD, EOMES, FN1, CALR, ENO1, HSPD1, HIST1H4A, PADI4, PTBP1, FEN1, DDX5, SFPQ, TUBB, LMNB1, NPM1, DDX21, FGB, ASMTL, SPP1, PDIA6, TNC, ACTB, IGF1, CPSF6, MCM2, DNAJB1, CLU, RBM39, IGFBP6, NONO, SRSF7, EIF4H, TNFSF13, CCL5, CD80, CSF2RA, IL6R, CD28, IFNA2, APOE_c, RBMS1_c, FGA_c, CXCL5_c, HNRNPA1_c, VIM_c, AEBP1_c, TRA2B_c, HIST1H2BD_c, EOMES_c, FN1_c, CALR_c, ENO1_c, HSPD1_c, HIST1H4A_c, PADI4_c, PTBP1_c, FEN1_c, DDX5_c, SFPQ_c, TUBB_c, LMNB1_c, NPM1_c, DDX21_c, FGB_c, ASMTL_c, SPP1_c, PDIA6_c, TNC_c, ACTB_c, IGF1_c, CPSF6_c, DNAJB1_c, CLU_c, RBM39_c, IGFBP6_c, NONO_c, SRSF7_c, EIF4H_c, DCTN1, CD86, NoGeneSymbol, NoGeneSymbol, CCL2, IL17RA, IL1B, DMTF1, DCTN1, CKAP4, IL2RG, C1QA, MS4A1, CHD4, MLF2, CCL21, VEGFB, S100A8, IL1A, MMP3, IL15, TNF, ALAS1, AK2, EHBP1, MED8, NOP56, EBNA1BP2, WDR44, UPF3B, SCFD1, ZPR1, RAB11FIP3, C1orf131, ROBO1, C15orf57, C1orf131, CXXC1, ADAMTS13, MRPL11, TNFRSF10B, SPEG, GSN, NREP, MPST, PPP1R9B, ACAA1, IL23A, IFNL3, CCL8, IL26, IL1F10, CXCL8, IFNL2, IL36RN, CCL14, IL25, IL18, IL37, IL22, IL21, IL27, IL36A, CCL7, IL17A, IL36G, CCL25, CCL11, IL33, ACAT2, OGT, KIDINS220, ERP29, UBXN11, KIF5A, HNRNPAB, SIGIRR, DHX34, BRMS1, PRKCSH, RPS28, STMN2, LRPAP1, DOT1L, SEC23B, SGTA, TIMM10B, GAREM, UBAC1, WDR34, SHF, RRM1, PPM1A, DAAM1, CCDC51, CTAG1B, SCAP, PYGB, RBM26, HCLS1, HDHD2, LGALSL, PPIG, CLIP1, PARP8, OSBP2, CRTAC1, USE1, HSBP1, DOCK6, RDH16, FAM60A, PER1, HIBCH, PDZK1, RFFL, FAM107A, ISCU, TRAF1, ATP5H, PPP1R15A, ITFG3, GON4L, KRT18, PRUNE2, GRP, DENND4B, MMP2, S100A9, MIF, CERS5, COL18A1, SRC.YES1, KRT7, MMP7, MUCL1, RBM28, LSR, SMYD2, TNFSF14, FGF21, RPS6KA3, WDR55, MTA1, PSMD10, MAZ, TPM1, PTN, RPL12, KLKB1, KRT19, MAGED2, COL3A1, TRAF3IP1, PLD3, IFNA1, NAP1L4, CCDC74A, FAHD2A.FAHD2B, CXCL1, CTSB, BAG3, CASP1, CASP7, MMP13, IFIH1, CASP10, KRT8, ATP6V1A, ZFAND2B, GLT8D1, IL2RA, KRT20, ERN1, IL12B, CRTAP, C4B, ARFIP2, COL4A5, IL1RL1, C8G, CXCL3, FAM104B, TRAK2, IFI44, IFNA6, IFNGR2, MMP17, C1QB, TNFRSF1B, C4A, IFIT3, CFB, IFNA10, C8B, IFNA4, GNPTG, LCN2, CFHR3, OLFM2, CTSD, TIMP1, ATXN7L3, CLCN2, CHID1, TRAF2, GAREML, CTSL, TLR2, PDCD6IP, PRAP1, OBSL1, TPP2, CTSW, ITIH1, COL4A2, OSTF1, TNFAIP1, CTSH, CRYBG3, CXCL2, CASP2, TNFAIP3, IL1RN, CHI3L1, IL16, IL1R1, IRF1, C17orf85, IL6ST, IL3, IRAK4, LAG3, PDGFB, IL10, IL4R, CCL4, PDGFA, IRF4, MMP28, EGR3, TELO2, LIAS.PRDX1, CASP8, KRT6B, CASP5, KRT6A, CTSV, MMP12, DDX24, PCSK1N, FBXO18, ZNF579, CTSK, USP48, CCDC136, IGFBP2, STMN4, TTLL12, HOMER3, FAM13A, NOVA2, C1QBP, SNRPA, SNRPC, SNRPB2, TROVE, TRIM21, CENPH, Control_EP, Control_BSA, Control_Ecoli500, Control_msIgG6000, Control_huIgG6000, Control_huIgG0600, Control_huIgG0060, Control_huIgG0006, BGN_c, NSRP1, SUMO3, VCAM1, EIF4G2, RNF8, C11orf30, NAA35, CHMP5, PHYHIP, GBA, GSPT2.

*Tables*

Supplementary Table 1. Associations between cluster membership and EULAR response at 3/6 months, adjusted for age, gender, disease duration and baseline DAS28.

| **Cluster** | **OR_adj_ (95% confidence intervals)** | **p-value** |
| --- | --- | --- |
| *Good EULAR response* | | |
| 1 | 0.83 (0.38 - 1.77) | 0.636 |
| 2 | 0.92 (0.37 – 2.21) | 0.859 |
| 3 | 1.63 (0.85-3.12) | 0.137 |
| 4 | 1.22 (0.70 – 2.13) | 0.477 |
| Cluster 1/2 | 0.86 (0.46 – 1.56) | 0.616 |
| Cluster 3/4 | 1.55 (095 – 2.53) | 0.077 |
| *Poor EULAR response* | | |
| 1 | 1.38 (0.60-3.20) | 0.445 |
| 2 | 1.67 (0.66 – 4.27) | 0.280 |
| 3 | 0.59 (0.27 – 1.24) | 0.173 |
| 4 | 0.79 (0.41 – 1.48) | 0.463 |
| Cluster 1/2 | 1.59 (0.82 – 3.10) | 0.166 |
| Cluster 3/4 | 0.63 (0.36 – 1.08) | 0.096 |

Supplementary Table 2. Multivariate model of differentially expressed autoantibodies in RA and improvement in DAS28 at 3/6 months, adjusted for age, gender, disease duration and baseline DAS28.

| *Protein* | *Coefficient* | *95% confidence interval* | *p-value* |
| --- | --- | --- | --- |
| ASMTL_c | 0.13 | -0.61-0.87 | 0.725679 |
| EIF4H_c | 0.57 | -0.05-1.19 | 0.072362 |
| SPP1_c | 0.24 | -0.41-0.90 | 0.469873 |
| NONO_c | 0.12 | -0.45-0.69 | 0.681208 |
| CLU_c | 0.58 | -0.14-1.31 | 0.121235 |
| VIM_c | -0.58 | -1.27-0.11 | 0.104079 |
| FN1_c | -0.58 | -1.20-0.05 | 0.075111 |
| CPSF6_c | -0.43 | -1.08-0.21 | 0.189360 |
| TRA2B_c | -0.30 | -0.95-0.35 | 0.366083 |
| RBMS1_c | -0.10 | -0.70-0.50 | 0.744288 |
| ACTB_c | 0.20 | -0.60-1.00 | 0.622549 |
| HNRNPA1_c | 0.69 | 0.05-1.34 | 0.036636* |
| DNAJB1_c | 0.60 | -0.05-1.24 | 0.072281 |
| TNC_c | -0.07 | -0.73-0.59 | 0.840570 |
| FGB_c | -0.21 | -1.02-0.60 | 0.612186 |
| SFPQ_c | 0.36 | -0.22-0.94 | 0.226020 |
| SRSF7_c | 0.12 | -0.64-0.87 | 0.762739 |
| TUBB_c | 0.29 | -0.41-0.99 | 0.415842 |
| PADI4_c | 0.16 | -0.82-1.13 | 0.753365 |
| AEBP1_c | -0.15 | -0.81-0.51 | 0.663236 |
| DDX5_c | 0.51 | -1.16-0.14 | 0.128602 |
| TNFSF13 | -0.02 | -0.66-0.62 | 0.957524 |
| APOE_c | -0.04 | -0.68-0.61 | 0.911108 |
| RBM39_c | 0.05 | -0.71-0.82 | 0.888700 |
| IL1B | 0.06 | -0.63-0.74 | 0.865323 |
| TUBB | -0.15 | -1.17-0.88 | 0.782191 |
| FGA_c | -0.31 | -1.09-0.47 | 0.438587 |
| IGF1_c | -0.33 | -1.17-0.51 | 0.438713 |
| FEN1_c | -0.12 | -0.89-0.64 | 0.752585 |
| ENO1_c | -0.31 | -0.97-0.34 | 0.352740 |
| MMP2 | -0.10 | -0.92-0.73 | 0.820763 |
| OBSL1 | -0.37 | -1.29-0.55 | 0.432406 |
| HIST1H4A_c | 0.66 | -0.42-1.75 | 0.231999 |
| PTBP1_c | -0.06 | -0.70-0.57 | 0.842166 |

AIC: 599.54

Supplementary Table 3. Multivariate model of differentially expressed autoantibodies in RA and good EULAR response at 3/6 months, adjusted for age, gender, disease duration and baseline DAS28.

| *Protein* | *OR_adj_* | *95% confidence interval* | *p-value* |
| --- | --- | --- | --- |
| ASMTL_c | 0.77 | 0.19-2.93 | 0.70342 |
| EIF4H_c | 1.37 | 0.46-4.19 | 0.57910 |
| SPP1_c | 1.92 | 0.57-6.82 | 0.29999 |
| NONO_c | 1.03 | 0.37-2.91 | 0.95876 |
| CLU_c | 3.32 | 0.87-13.70 | 0.08551 |
| VIM_c | 0.22 | 0.05-0.81 | 0.02992* |
| FN1_c | 0.38 | 0.11-1.18 | 0.10403 |
| CPSF6_c | 1.26 | 0.38-4.24 | 0.70196 |
| TRA2B_c | 0.80 | 0.24-2.61 | 0.71040 |
| RBMS1_c | 0.97 | 0.31-2.90 | 0.95181 |
| ACTB_c | 1.98 | 0.47-8.65 | 0.35496 |
| HNRNPA1_c | 1.76 | 0.56-5.73 | 0.33729 |
| DNAJB1_c | 1.75 | 0.56-5.57 | 0.33414 |
| TNC_c | 1.00 | 0.31-3.19 | 0.99962 |
| FGB_c | 2.19 | 0.52-9.78 | 0.29043 |
| SFPQ_c | 1.46 | 0.53-4.05 | 0.46020 |
| SRSF7_c | 0.87 | 0.22-3.38 | 0.84082 |
| TUBB_c | 1.49 | 0.42-5.61 | 0.54174 |
| PADI4_c | 0.86 | 0.15-5.11 | 0.86387 |
| AEBP1_c | 0.64 | 0.19-2.07 | 0.46186 |
| DDX5_c | 0.42 | 0.13-1.31 | 0.14536 |
| TNFSF13 | 1.88 | 0.61-5.88 | 0.26678 |
| APOE_c | 1.06 | 0.33-3.34 | 0.92147 |
| RBM39_c | 0.76 | 0.19-2.81 | 0.68715 |
| IL1B | 0.55 | 0.15-1.93 | 0.35600 |
| TUBB | 0.64 | 0.09-4.33 | 0.65325 |
| FGA_c | 0.35 | 0.07-1.52 | 0.17802 |
| IGF1_c | 0.39 | 0.08-1.66 | 0.21589 |
| FEN1_c | 1.10 | 0.28-4.39 | 0.88763 |
| ENO1_c | 1.10 | 0.33-3.55 | 0.87709 |
| MMP2 | 0.99 | 0.21-4.77 | 0.99033 |
| OBSL1 | 1.01 | 0.20-5.37 | 0.88211 |
| HIST1H4A_c | 7.09 | 0.97-63.06 | 0.06334 |
| PTBP1_c | 0.54 | 0.14-1.81 | 0.33161 |

AIC: 267.63

Supplementary Table 4. Multivariate model of differentially expressed autoantibodies in RA and poor EULAR response at 3/6 months, adjusted for age, gender, disease duration and baseline DAS28.

| *Protein* | *OR_adj_* | *95% confidence interval* | *p-value* |
| --- | --- | --- | --- |
| ASMTL_c | 1.12 | 0.26-4.98 | 0.874497 |
| EIF4H_c | 0.62 | 0.18-2.09 | 0.436394 |
| SPP1_c | 0.38 | 0.11-1.44 | 0.154885 |
| NONO_c | 0.50 | 0.16-1.52 | 0.228854 |
| CLU_c | 0.56 | 0.12-2.57 | 0.456928 |
| VIM_c | 4.19 | 1.07-18.32 | 0.046355* |
| FN1_c | 2.43 | 0.69-9.36 | 0.177421 |
| CPSF6_c | 1.14 | 0.31-4.17 | 0.847322 |
| TRA2B_c | 0.97 | 0.27-3.60 | 0.962122 |
| RBMS1_c | 1.47 | 0.45-5.04 | 0.527093 |
| ACTB_c | 1.08 | 0.21-5.56 | 0.922595 |
| HNRNPA1_c | 0.72 | 0.19-2.58 | 0.609838 |
| DNAJB1_c | 0.41 | 0.11-1.48 | 0.176442 |
| TNC_c | 0.40 | 0.09-1.64 | 0.211766 |
| FGB_c | 0.98 | 0.19-5.09 | 0.981770 |
| SFPQ_c | 0.73 | 0.24-2.21 | 0.569642 |
| SRSF7_c | 1.09 | 0.23-4.88 | 0.915449 |
| TUBB_c | 0.64 | 0.15-2.55 | 0.528758 |
| PADI4_c | 0.43 | 0.05-3.66 | 0.443270 |
| AEBP1_c | 1.35 | 0.37-4.94 | 0.645107 |
| DDX5_c | 3.00 | 0.87-1.13 | 0.088936 |
| TNFSF13 | 0.88 | 0.26-3.04 | 0.842396 |
| APOE_c | 1.06 | 0.30-3.66 | 0.928080 |
| RBM39_c | 1.09 | 0.25-4.71 | 0.908613 |
| IL1B | 1.05 | 0.26-4.31 | 0.941550 |
| TUBB | 2.29 | 0.29-1.97 | 0.433853 |
| FGA_c | 4.44 | 0.86-2.61 | 0.082382 |
| IGF1_c | 4.04 | 0.86-2.30 | 0.089396 |
| FEN1_c | 1.25 | 0.25-6.06 | 0.779424 |
| ENO1_c | 0.98 | 0.27-3.57 | 0.973932 |
| MMP2 | 0.51 | 0.09-2.85 | 0.448026 |
| OBSL1 | 2.37 | 0.40-13.90 | 0.333026 |
| HIST1H4A_c | 0.11 | 0.01-1.01 | 0.062201 |
| PTBP1_c | 1.68 | 0.47-6.55 | 0.432187 |

AIC: 249.27
